# A Linear City Model with Asymmetric Consumer Distribution

**DOI:** 10.1371/journal.pone.0129068

**Published:** 2015-06-02

**Authors:** Ofer H. Azar

**Affiliations:** Department of Business Administration, Ben-Gurion University of the Negev, Beer Sheva, Israel; Middlesex University London, UNITED KINGDOM

## Abstract

The article analyzes a linear-city model where the consumer distribution can be asymmetric, which is important because in real markets this distribution is often asymmetric. The model yields equilibrium price differences, even though the firms’ costs are equal and their locations are symmetric (at the two endpoints of the city). The equilibrium price difference is proportional to the transportation cost parameter and does not depend on the good's cost. The firms' markups are also proportional to the transportation cost. The two firms’ prices will be equal in equilibrium if and only if half of the consumers are located to the left of the city’s midpoint, even if other characteristics of the consumer distribution are highly asymmetric. An extension analyzes what happens when the firms have different costs and how the two sources of asymmetry – the consumer distribution and the cost per unit – interact together. The model can be useful as a tool for further development by other researchers interested in applying this simple yet flexible framework for the analysis of various topics.

## Introduction

The linear-city model, introduced in the seminal article of Hotelling [1], has become a very useful tool for the analysis of competition with product (or seller) differentiation. Various issues have been analyzed using the linear-city model, including for example add-on pricing [2], preference for variety [3], and pricing of multi-product firms [4]. The usual linear-city model assumes symmetric and uniform distribution of consumers over the city line. It seems interesting, however, to develop a model in which consumer distribution does not have a specific functional form (e.g., a uniform distribution) and can also be asymmetric, for several reasons.

First, in real markets asymmetries in consumer demand between competing firms often exist and it is therefore interesting to explore how these will affect competition in the linear-city framework. For example, in a city where one side is more residential and the other side more industrial (but also with consumers, either ones who live there or those who work there), a store located in the residential area is more conveniently located than a store in the industrial area for most consumers, but not all of them. That is, the preference of consumers between the two stores is asymmetric, but not to the extent that everyone prefers one seller, as in vertical differentiation. To analyze the competition between such firms we can use the linear city model but we have to introduce asymmetry in the consumer distribution over the city line.

Second, a model that yields tractable and reasonable results for any consumer distribution can be a useful tool and become a basic model that other researchers can further develop to analyze various topics of interest. An essential part of such a tool is that the model is simple, so that when others add to it complexities of the issues they want to analyze, the model is still solvable and understandable. Therefore I tried to keep the model presented below as simple as possible. The exception is that the consumer distribution can take any form, which complicates the model but is an important part.

Third, an important topic in economics is price dispersion, a phenomenon that contradicts the law of one price and raises the question why consumers buy from a store when another store sells the same good for a lower price. A short literature review [5] writes about price dispersion that “It is an important topic in the field of the economics of information in that there is considerable empirical evidence that price dispersion is widespread and significant. Yet, it has proven surprisingly difficult for economists to derive satisfactory models that support price dispersion as an equilibrium phenomenon.”

Several theoretical models analyze price dispersion. Reinganum [6] presents a model where identical buyers with elastic demand sample sequentially at a cost from a known price distribution. Given the distribution of the firms’ marginal costs, they behave as monopolistic competitors, and this results in a distribution of prices. Fershtman [7] studies oligopolistic price competition under the assumption that consumers do not respond to small price differences and shows that this can yield an equilibrium where firms do not charge the same price. Rob [8] shows that depending on the nature of search costs, the long-run equilibria may involve single, multi- and continuous price distributions, and provides a method for computing equilibrium price distributions. Caplin and Nalebuff [9] study price competition among differentiated products, and provide conditions that guarantee a pure-strategy price equilibrium for any number of firms producing any set of products. Polo [10] analyzes duopoly price competition with perfectly and imperfectly informed consumers who exist together. When some consumers do not observe prices, the equilibrium can involve price dispersion, and price tends to rise above the full-information price. Janssen and Moraga-Gonzalez [11] examine an oligopoly model where some consumers engage in costly non-sequential search to reveal prices. They find three price-dispersed equilibria with low, moderate and high search intensity. Increasing the number of firms affects welfare, expected prices, search behavior and price dispersion in a manner that is sensitive to the equilibrium consumers' search intensity and to the status quo number of firms. Duopoly yields identical expected price and price dispersion but higher welfare than an infinite number of firms. Anderson and De Palma [12] study pricing when consumers follow reservation-price rules and obtain price dispersion in pure strategies even when marginal costs are the same for all firms. They also find that the range of price dispersion increases with the number of firms, and that when prices are chosen sequentially the equilibrium pricing pattern is the same. Sinitsyn [13] presents a model of sales with product differentiation and heterogeneity in consumer preferences and shows that in the mixed strategy equilibrium firms choose the prices from a finite set and do not choose a continuum of prices. Chudik [14] develops a stock-flow matching model with fully-informed participants and prices that are set ex-ante. When sellers commit to sell at an advertised price, the unique equilibrium is characterized by price dispersion. Backus et al. [15] develop a model of competing second-price auctions that captures price dispersion, and then provide evidence on search costs and price dispersion using data from eBay. The literature on price dispersion also includes empirical studies [16–18] and experimental studies [19], but these are less related to the current paper.

The literature often explains price dispersion as coming from costly search (consumers do not know all prices and have to incur costs to find them out). However, equilibrium price differences can also be the result of seller differentiation (e.g., a geographical differentiation of sellers’ locations). Because the linear-city models in the literature usually assume a uniform and symmetric consumer distribution, however, they do not yield equilibrium price differences (if the firms' costs are identical).

A few papers analyze consumer distribution that is not uniform. Shilony [20] finds that for any distribution if the two firms are located too close to each other, an equilibrium does not exist. Tabuchi and Thisse [21] study consumer concentration around the market center and obtain asymmetric equilibria. Anderson, Goeree and Ramer [22] allow the consumer density to be log-concave in a two-stage model with location choice and then price competition. They find that equilibrium locations are closer and prices lower when the density is tighter. Benassi and Chirco [23] find a sufficient condition for the existence of multiple asymmetric equilibria when the consumer density is symmetric, in terms of a lower bound on the Gini coefficient of the preference distribution.

The current article adds to the literature by presenting a linear-city model that allows asymmetric consumer distribution without assuming any particular functional form of this distribution, and yields equilibrium price difference in a simple framework. The equilibrium price difference in the model is not the result of costly search or cost differences, as in many other price dispersion models, but rather a result of seller location differentiation combined with asymmetry in the consumer distribution. The article reports the equilibrium prices, markups and price difference, and examines how they depend on the consumer distribution, the transportation cost parameter, and the good's cost. Analyzing the case of asymmetric consumer distribution is important because in real markets this distribution can often be asymmetric. Despite letting the consumer distribution take any form including complex distributions, the model produces some elegant and simple results. The model yields equilibrium price difference and thus shows that the linear-city framework can be used to analyze equilibrium price differences even when the firms’ costs and locations are symmetric. The results suggest that the equilibrium price difference is proportional to the transportation cost parameter and does not depend on the good's cost. The firms' markups are also proportional to the transportation cost. The two firms’ prices are equal in equilibrium if and only if half of the consumers are located to the left of the city’s midpoint, even if other characteristics of the consumer distribution are highly asymmetric. Finally, an extension analyzes the case of firms that have different costs, together with an asymmetric consumer distribution. The simplicity of the model on one hand, and the full flexibility that it permits for the consumer distribution on the other hand, suggest that it can be useful as a tool for further development, using its framework as the basis for more complex models that analyze additional topics.

## The Model

A linear city with consumers whose mass is normalized to 1 is served by two price-competing firms, which are located at the endpoints (firm 1 at 0 and firm 2 at 1). The mass of consumers between 0 and any point *b* is *F*(*b*), and the PDF of consumers is denoted by *f*(*b*) = *F’*(*b*), where *f*(*b*) is potentially asymmetric. Obviously, *F* satisfies *F*(0) = 0 and *F*(1) = 1. I assume that the support of *f*(*b*) is the entire interval [0, 1], and that *F* is twice continuously differentiable. The distance between the consumer and the firm can represent either physical distance the consumer has to travel, or the difference between the consumer’s preferences and the firm’s characteristics, and is an added cost from the consumer perspective, referred to as the transportation costs. Following much of the literature, I assume quadratic transportation costs (the results, however, are essentially the same also with linear transportation costs, see the footnote in the proof of Proposition 1), so the total cost a consumer located at *d* incurs is *p*_*1*_ *+ td* ^2^ when purchasing from firm 1 and *p*_*2*_ *+ t*(1-*d*)^2^ when buying from firm 2, where *t* is a parameter that represents the magnitude of the transportation costs. Every consumer buys one unit of the good from the firm that offers the lower total cost, if this total cost is smaller than *U* (i.e., *U* is the good’s value for the consumer if he and the firm share the same location).

I assume that *U* is high enough that in equilibrium everyone buys from one of the firms. A sufficient condition for everyone to buy in equilibrium is that *U* > min [*t* + min (*p*_1_, *p*_2_), *t*/4 + max (*p*_1_, *p*_2_)]. The reason is that if consumers buy from the cheapest firm, their total cost never exceeds *t* + min (*p*_1_, *p*_2_), and if they buy from the closest firm, it never exceeds 0.5^2^ *t* + max (*p*_1_, *p*_2_). This assumption that the market is covered is a common assumption in linear-city type models and it implies that there are no consumers who are on the boundary between buying and not buying at all. A firm that increases prices, however, can lose its customers to its competitor and therefore each firm still faces a demand function with a negative slope even though the total number of units sold in the market is fixed. Assuming instead that *U* is small enough that in equilibrium some consumers may not buy from any firm will complicate the analysis and go against the purpose of this model to offer a simple framework to analyze a duopoly with asymmetric consumer distribution. Moreover, in such more complex model, if the value of *U* is not binding in equilibrium, we are back at the current assumption and analysis. If *U* is binding (some consumers do not buy at all in equilibrium), the price is equal to the arbitrary *U* assumed, and this does not seem to offer interesting insights about the firms' pricing decisions. In addition, if *U* is binding in equilibrium it means that we have two local monopolies, each with a captive market, whereas the purpose of this paper is to model duopoly competition and not monopoly pricing or the transition between duopoly and monopoly.

I further assume that the firms have a constant marginal cost of *c* per unit. Proposition 1A characterizes the equilibrium prices and Proposition 1B analyzes the sufficient and necessary second-order conditions.

### Proposition 1

A. Equilibrium prices, denoted by *p*_1_* and *p*_2_*, satisfy the following two equations:

$$p_{1}^{*}=c_{}^{}+\frac{2tF\left(\frac{p_{2}^{*}-p_{1}^{*}}{2t}+\frac{1}{2}\right)}{f\left(\frac{p_{2}^{*}-p_{1}^{*}}{2t}+\frac{1}{2}\right)}$$

and

$$p_{2}^{*}=c_{}+\frac{2t\left[1-F\left(\frac{p_{2}^{*}-p_{1}^{*}}{2t}+\frac{1}{2}\right)\right]}{f\left(\frac{p_{2}^{*}-p_{1}^{*}}{2t}+\frac{1}{2}\right)}.$$

B. The sufficient second-order conditions for *p*_1_* and *p*_2_* to constitute an equilibrium are (both have to hold simultaneously): $\frac{f\prime \left(\frac{p_{2}^{*}-p_{1}^{}}{2t}+\frac{1}{2}\right)(p_{1}-c)}{4t}-f\left(\frac{p_{2}^{*}-p_{1}^{}}{2t}+\frac{1}{2}\right)<0$ for all *p*_1_ϵ(max(*c*, *p*_2_*- *t*), *p*_2_*+ *t*), and $\frac{-f\prime \left(\frac{p_{2}^{}-p_{1}^{*}}{2t}+\frac{1}{2}\right)(p_{2}-c)}{4t}-f\left(\frac{p_{2}^{}-p_{1}^{*}}{2t}+\frac{1}{2}\right)<0$ for all *p*_2_ϵ(max(*c*, *p*_1_*- *t*), *p*_1_*+ *t*).

Often, however, *p*_1_* and *p*_2_* are an equilibrium even though the conditions above are not met. A necessary second-order condition for *p*_1_* and *p*_2_* to be the equilibrium prices is:

$$2{\left[f\left(\frac{p_{2}^{*}-p_{1}^{*}}{2t}+\frac{1}{2}\right)\right]}^{2}\geq \text{max}\left\{F\left(\frac{p_{2}^{*}-p_{1}^{*}}{2t}+\frac{1}{2}\right)f\prime \left(\frac{p_{2}^{*}-p_{1}^{*}}{2t}+\frac{1}{2}\right),\left[F\left(\frac{p_{2}^{*}-p_{1}^{*}}{2t}+\frac{1}{2}\right)-1\right]f\prime \left(\frac{p_{2}^{*}-p_{1}^{*}}{2t}+\frac{1}{2}\right)\right\}$$

#### Proof

A. Since consumers’ total cost is increasing in their distance from the firm from which they buy, there is a unique location of an indifferent consumer, denoted by *x*, where *x* satisfies *p*_1_ + *tx* ^2^ = *p*_2_ + *t*(1 − *x*)^2^. Simple algebra then yields *x* = (*p*_2_ - *p*_1_)/2*t* + 0.5. Notice that with linear transportation costs the indifferent consumer is defined by the equation *p*_1_ + *tx* = *p*_2_ + *t*(1-*x*), which also results in *x* = (*p*_2_ − *p*_1_)/2*t* + 0.5. This implies that the entire analysis in this section is exactly the same if we assume linear (rather than quadratic) transportation costs. The only difference is that now the sufficient condition that ensures that everyone buys in equilibrium becomes *U* > min [*t* + min (*p*_1_, *p*_2_), *t*/2 + max (*p*_1_, *p*_2_)] rather than *U* > min [*t* + min (*p*_1_, *p*_2_), *t*/4 + max (*p*_1_, *p*_2_)].

The quantity sold by firm 1 is *F*(*x*) and firm 2's quantity is 1 − *F*(*x*). Firm 1’s profits are therefore given by (*p*_1_ − *c*)*F*(*x*) (notice that *x* is a function of *p*_1_ and *p*_2_ and the firms do not treat it as exogenous). Firm 1 maximizes its profits by choosing *p*_1_. The first-order condition for profit maximization is given by:

$F(x)-\frac{p_{1}^{*}-c}{2t}f(x)=0,$ from which it follows that $p_{1}^{*}=c+\frac{2tF(x)}{f(x)}.$ Similarly, profit maximization by firm 2 implies: $1-F(x)-\frac{p_{2}^{*}-c}{2t}f(x)=0,$ from which it follows that $p_{2}^{*}=c+\frac{2t[1-F(x)]}{f(x)}.$

Substituting *x* = (*p*_2_ - *p*_1_)/2*t* + 0.5 in these expressions yields the values of *p*_1_* and *p*_2_* in Proposition 1.

B. **Second-order sufficient conditions**: For *p*_1_* and *p*_2_* to be equilibrium prices, a sufficient condition is that firm 1’s profit is strictly concave in *p*_1_ given the price *p*_2_*, and similarly for firm 2. This has to be true for all relevant prices. For firm 1, we can limit attention to prices that are strictly above *c*, weakly above *p*_2_* − *t*, and strictly below *p*_2_* + *t*. Firm 1 can always make positive profits by choosing *p*_1_ = *c* + ε where ε → 0, so it is clear why *p*_1_ > *c*. Also, for any *p*_1_ ≥ *p*_2_* + *t* the demand facing firm 1 is zero and so it makes zero profits, so we can limit attention to *p*_1_ < *p*_2_* + t. Finally, for any *p*_1_ ≤ *p*_2_* − *t*, firm 1 has the same demand (all the consumers, or a mass of 1), so it never has an incentive to reduce its price below *p*_2_* − *t*. To summarize, the strict concavity only has to hold for *p*_1_ϵ(max(*c*, *p*_2_*− *t*), *p*_2_*+ *t*). By similar arguments, strict concavity of firm 2’s profit in *p*_2_ has to hold only for *p*_2_ϵ(max(*c*, *p*_1_*− *t*), *p*_1_*+ *t*). After some simplification, the second-order sufficient conditions become: $\frac{f\prime \left(\frac{p_{2}^{*}-p_{1}^{}}{2t}+\frac{1}{2}\right)(p_{1}-c)}{4t}-f\left(\frac{p_{2}^{*}-p_{1}^{}}{2t}+\frac{1}{2}\right)<0$ for all *p*_1_ϵ(max(*c*, *p*_2_*− *t*), *p*_2_*+ *t*), and $\frac{-f\prime \left(\frac{p_{2}^{}-p_{1}^{*}}{2t}+\frac{1}{2}\right)(p_{2}-c)}{4t}-f\left(\frac{p_{2}^{}-p_{1}^{*}}{2t}+\frac{1}{2}\right)<0$ for all *p*_2_ϵ(max(*c*, *p*_1_*− *t*), *p*_1_*+ *t*).

#### Second-order necessary conditions

The second-order necessary conditions are that each profit function is weakly concave in the same firm's price when evaluated at the candidate equilibrium (*p*_1_* and *p*_2_*). This yields:

$$\frac{\partial^{2}\pi_{1}}{\partial p_{1}{}^{2}}(p_{1}^{*},p_{2}^{*})=\frac{1}{t}\left[\frac{\left(p_{1}^{*}-c)f\prime (x\right)}{4t}-f(x)\right]\leq 0,$$

and

$$\frac{\partial^{2}\pi_{2}}{\partial p_{2}{}^{2}}(p_{1}^{*},p_{2}^{*})=\frac{1}{t}\left[-\frac{\left(p_{2}^{*}-c)f\prime (x\right)}{4t}-f(x)\right]\leq 0.$$

Substituting for (*p*_1_* − *c*) and (*p*_2_* − *c*) from the first-order conditions and simplifying we then get:

$$\frac{\partial^{2}\pi_{1}}{\partial p_{1}{}^{2}}(p_{1}^{*},p_{2}^{*})=\frac{F(x)f\prime (x)-2{\left[f(x)\right]}^{2}}{2f(x)}\leq 0,$$

and

$$\frac{\partial^{2}\pi_{2}}{\partial p_{2}{}^{2}}(p_{1}^{*},p_{2}^{*})=\frac{-[1-F(x)]f\prime (x)-2{\left[f(x)\right]}^{2}}{2f(x)}\leq 0.$$

Since *f*(*x*) > 0, combining the two inequalities yields: 2[*f*(*x*)]^2^ ≥ max {*F*(*x*)*f* ‘(*x*), [*F*(*x*) - 1]*f* ‘(*x*)}, from which the condition in the proposition follows immediately by substituting *x* = (*p*_2_ - *p*_1_)/2*t* + 0.5.

In the rest of the analysis I assume that the second-order conditions are satisfied, and therefore equilibrium prices are as mentioned in Proposition 1A. The location of the indifferent consumer is denoted by *x* = (*p*_2_* - *p*_1_*)/2*t* + 0.5, and I use Δ to denote the price difference (∆ ≡ *p*_2_* - *p*_1_*) to simplify notation. Proposition 2 describes how the price difference depends on *t*:

### Proposition 2

In equilibrium, ∆ = β*t* for some constant β (which depends only on *F* and is independent of *c*).

#### Proof

Using the results from Proposition 1, the price difference is given by:

$$\Delta (t)=\frac{2t[1-2F(x)]}{f(x)}=\frac{2t\left[1-2F\left(\frac{\Delta (t)}{2t}+\frac{1}{2}\right)\right]}{f\left(\frac{\Delta (t)}{2t}+\frac{1}{2}\right)}.$$

Differentiate both sides with respect to *t* and rearrange to obtain:

$$\frac{f{\left(x\right)}^{2}\Delta \prime (t)}{2}=\left\{1-2F(x)-\frac{f(x)[t\Delta \prime (t)-\Delta (t)]}{t}\right\}f(x)-t[1-2F(x)]f\prime (x)\frac{t\Delta \prime (t)-\Delta (t)}{2t^{2}}$$

Further algebraic manipulations yield:

$$\Delta \prime (t)=\frac{\left[1-2F(x)+\frac{f(x)\Delta (t)]}{t}\right]f(x)+\frac{\left[1-2F(x)]f\prime (x)\Delta (t\right)}{2t^{}}}{\left\{\frac{3f{\left(x\right)}^{2}}{2}+\frac{\left[1-2F(x)]f\prime (x\right)}{2}\right\}}$$

Some additional algebra, using $\Delta (t)=\frac{2t[1-2F(x)]}{f(x)}$, yields the result that Δ’(*t*) = Δ(*t*)/*t*. Taking the derivative of both sides with respect to *t* yields Δ”(*t*) = [*t*Δ’(*t*) − Δ(*t*)]/*t* ^2^ = 0, from which it follows that Δ(*t*) is an affine function of *t*. Substituting *t* = 0 in *t*Δ’(*t*) − Δ(*t*) = 0 implies that Δ(0) = 0 and it follows that Δ(*t*) = β*t* for some constant β.

Despite not assuming any particular functional form for the consumer distribution, we obtain strong results about the equilibrium price difference, Δ: it is proportional to *t*; and it does not depend on the good's cost, *c*. This shows the importance of *t*, which is a measure of product differentiation, in shaping the equilibrium. A higher *t* increases the price dispersion, and does so in a linear way. On the other hand, we also see that the cost per unit to the firms does not play a role in determining the equilibrium price difference: *c* does not affect Δ. The price difference is created due to product differentiation or geographical differentiation of consumers and the cost, which is equal for both firms, does not affect the level of differentiation and therefore also not the price difference.

It is interesting to see in what situations equilibrium prices will be equal. For example, can we have an asymmetric consumer distribution that still yields equal prices? Under what conditions this happens? Proposition 3 reports the condition that ensures that equilibrium prices are equal.

### Proposition 3

The prices of the two firms are equal if and only if exactly half of the consumers are located to the left of the linear city center, i.e., *F*(0.5) = 0.5.

#### Proof

Substitute Δ(*t*) = 0 in the expression for Δ(*t*) derived in the proof of Proposition 2:

$$\Delta (t)=\frac{2t[1-2F(x)]}{f(x)}=\frac{2t\left[1-2F\left(\frac{\Delta (t)}{2t}+\frac{1}{2}\right)\right]}{f\left(\frac{\Delta (t)}{2t}+\frac{1}{2}\right)}.$$

This gives 0 = 2*t*[1-2*F*(0.5)]/*f*(0.5). It follows that *F*(0.5) = 0.5.

This is an interesting result. We can have a consumer distribution that is heavily asymmetric and far from the uniform distribution, but if half of the consumers are located to the left of the city’s midpoint, the resulting price equilibrium will be symmetric with the same price charged by both firms. One simple condition tells us, regardless of the other characteristics of the consumer distribution, whether in equilibrium we have a price difference or not. When prices are equal, the indifferent consumer is located exactly at the midpoint of the city, i.e., *x* = 0.5 (see the proof of Proposition 1). This means that the transportation costs that could be obtained given the distribution of consumers are minimized, because each consumer buys from the closest firm. Since transportation costs are the only factor that reduces social welfare in the model (prices only represent a transfer from consumers to the firms), this also maximizes social welfare.

### Proposition 4

The quantity sold by each firm does not depend on *t* or *c*. In particular, *q*_*1*_ *= F* [(β + 1)/2] and *q*_2_ = 1 *F* [(β + 1)/2].

#### Proof

Substituting Δ(*t*) = *p*_2_* − *p*_1_* = β*t* into *x* = (*p*_2_* - *p*_1_*)/2*t* + 0.5 yields *x* = (β + 1)/2, from which it follows that *q*_*1*_ *= F* [(β + 1)/2] and *q*_2_ = 1 *F* [(β + 1)/2]. The quantities sold by the firms do not depend on *t* or *c* because β is a constant. The value of β (for a given consumer distribution) can be found by solving simultaneously the two equations that define *p*_1_* and *p*_2_* in Proposition 1 and then using β = Δ/*t* = (*p*_2_* - *p*_1_*)/*t*.

The intuition why quantities are not a function of *t* is the following: for the indifferent consumer (whose location is *x*), the cheaper firm must be the more remote one (otherwise he has strict preference and not indifference). When *t* increases, it becomes more costly to travel the greater distance to the more remote firm, but also the price difference becomes higher, and by exactly the same proportion. Consequently, the two effects exactly cancel each other and the location of the indifferent consumer does not change (and therefore also the quantities sold by the two firms remain unchanged).

Proposition 5 states another result, regarding the firms' markups. It shows once again the importance of the transportation cost parameter is shaping the equilibrium prices.

### Proposition 5

The markups of the firms, *p*_1_* − *c* and *p*_2_* − *c*, are proportional to *t*.

#### Proof

Substitute x = (β + 1)/2 (see Proposition 4) in the expressions for the two markups (from Proposition 1) to get:

$p_{1}^{*}-c=\frac{2tF\left(\frac{\beta +1}{2}\right)}{f\left(\frac{\beta +1}{2}\right)}$ and $p_{2}^{*}-c=\frac{2t\left[1-F\left(\frac{\beta +1}{2}\right)\right]}{f\left(\frac{\beta +1}{2}\right)}$. Because β is a constant, it follows that the markups are proportional to *t*.

### Numerical Analysis for Consumer Distribution *F(b) = b* ^*α*^

To give a more concrete idea about the specific values of possible equilibria of this model, the case of the consumer distribution *F*(*b*) = *b* ^α^ was analyzed in more detail. Table 1 presents the results of numerical analysis of the equilibrium for *F*(*b*) = *b* ^α^, for various values of α.

**Table 1:** Equilibrium for *F*(*b*) = *b* ^α^ and various values of α.

Α	Δ	*x*	*t*	*c*	*p*_1_*	*p*_2_*	*p*_1_*-*c*	*p*_2_*-*c*	π_1_	π_2_	π_1_+ π_2_	TC	Min TC
0.5	-0.341	0.330	1	1	2.319	1.978	1.319	0.978	0.757	0.416	1.174	0.085	0.062
0.6	-0.249	0.375	1	1	2.251	2.001	1.251	1.001	0.695	0.445	1.140	0.082	0.068
0.7	-0.172	0.414	1	1	2.183	2.010	1.183	1.010	0.638	0.465	1.103	0.080	0.074
0.8	-0.106	0.447	1	1	2.117	2.011	1.117	1.011	0.586	0.480	1.067	0.080	0.078
0.9	-0.049	0.475	1	1	2.056	2.007	1.056	1.007	0.541	0.491	1.032	0.082	0.081
1	0.000	0.500	1	1	2.000	2.000	1.000	1.000	0.500	0.500	1.000	0.083	0.083
1.25	0.099	0.550	1	1	1.879	1.979	0.879	0.979	0.416	0.516	0.932	0.089	0.087
1.5	0.174	0.587	1	1	1.783	1.957	0.783	0.957	0.352	0.527	0.879	0.096	0.087
1.75	0.233	0.617	1	1	1.705	1.938	0.705	0.938	0.302	0.535	0.838	0.102	0.086
2	0.281	0.640	1	1	1.640	1.921	0.640	0.921	0.263	0.543	0.806	0.107	0.083
2.5	0.354	0.677	1	1	1.541	1.895	0.541	0.895	0.204	0.558	0.762	0.115	0.076
3	0.408	0.704	1	1	1.469	1.877	0.469	0.877	0.164	0.571	0.735	0.119	0.069
5	0.536	0.768	1	1	1.307	1.843	0.307	0.843	0.082	0.618	0.700	0.122	0.042
10	0.671	0.836	1	1	1.167	1.839	0.167	0.839	0.028	0.699	0.727	0.101	0.015
50	0.875	0.937	1	1	1.037	1.912	0.037	0.912	0.001	0.876	0.878	0.034	0.001
100	0.923	0.962	1	1	1.019	1.943	0.019	0.943	0.000	0.924	0.924	0.018	0.000
2	0.281	0.640	1	1	1.640	1.921	0.640	0.921	0.263	0.543	0.806	0.107	0.083
2	0.562	0.640	2	1	2.281	2.842	1.281	1.842	0.525	1.087	1.612	0.107	0.083
2	0.281	0.640	1	2	2.640	2.921	0.640	0.921	0.263	0.543	0.806	0.107	0.083

The top part shows the equilibrium for the CDF *F*(*b*) = *b* ^α^ for various values of α, fixing *c* = 1 and *t* = 1. The analysis is for α ≥ 0.5 because this ensures that *p*_1_* and *p*_2_* are indeed equilibrium prices. The bottom three lines show how changing *t* or *c* affect the equilibrium, fixing α = 2. We can see that multiplying *t* by 2 also multiplies markups, profits, and the price difference (Δ) by 2, leaving the location of the indifferent consumer unchanged. Increasing *c* by 1 increases prices also by 1, leaving markups, profits, and the location of the indifferent consumer unchanged.

The standard linear city model which assumes uniform distribution of consumers corresponds to the case α = 1.

TC is total transportation costs (with quadratic transportation costs), which is equal to:

$$\overset{x}{\underset{0}{\int }}b^{2}f\left(b\right)db+\overset{1}{\underset{x}{\int }}{\left(1-b\right)}^{2}f\left(b\right)db.$$

Substituting *f*(*b*) = α*b* ^α −1^ we then obtain:

$$\overset{x}{\underset{0}{\int }}\alpha b^{\alpha +1}db+\overset{1}{\underset{x}{\int }}\left(1-2b+b^{2}\right)\alpha b^{\alpha -1}db.$$

It is easy to show that this expression is equal to:

$$1+\frac{\alpha }{\alpha +2}-x^{\alpha }+\frac{2\alpha }{\alpha +1}\left(x^{\alpha +1}-1\right).$$

The column Min TC presents the value of this expression when *x* = 0.5. This is the minimum transportation costs that could be obtained given the distribution of consumers; this minimum is obtained when each consumer buys from the closest firm (i.e., when *x* = 0.5). A social planner who maximizes welfare would like to implement *x* = 0.5 (for example by imposing on the two firms to choose the same price), because transportation costs are the only social cost in the model (by assumption everyone buys one unit in equilibrium, and the prices only represent a transfer from consumers to the firms and therefore have no welfare effects). The difference between TC and Min TC is therefore a social cost that results from the asymmetric distribution of consumers and from market forces.

### Asymmetric Costs and Consumer Distribution *F(b) = b* ^*2*^

An interesting question is what happens to the equilibrium price difference when we add another source of asymmetry to the model, in the form of asymmetric costs. Firm 1 incurs a cost of *c*_1_ per unit sold whereas firm 2 incurs a cost of *c*_2_ per unit sold. To provide tractable analysis despite the added complexity of asymmetric costs, I focus here on a specific functional form of the consumer distribution, namely *F*(*b*) = *b* ^2^. Notice that this means that *F*(0.5) = 0.25, which implies that 75% of the consumers are closer to firm 2 than to firm 1. This gives firm 2 an advantage, and in the absence of cost asymmetries it would result in a higher price and a higher profit for firm 2, as we have seen in Table 1. The cost asymmetry can either reinforce or alleviate the advantage of firm 2, depending on which firm has a lower cost. Before we proceed, let us first define *z* as the cost difference between firm 2 and firm 1:

$$z=c_{2}-c_{1}.$$

Proposition 6 characterizes the equilibrium price difference.

### Proposition 6

The equilibrium price difference is one of the two values that solve the following equation:

$$p_{2}^{*}-p_{1}^{*}=\frac{z-3t\pm \sqrt{17t^{2}+2zt+z^{2}}}{4}$$

#### Proof

Following a proof that is almost identical to that of Proposition 1 we obtain that equilibrium prices, denoted by *p*_1_* and *p*_2_*, satisfy the following two equations:

$$p_{1}^{*}=c_{1}^{}+\frac{2tF\left(\frac{p_{2}^{*}-p_{1}^{*}}{2t}+\frac{1}{2}\right)}{f\left(\frac{p_{2}^{*}-p_{1}^{*}}{2t}+\frac{1}{2}\right)}$$

and

$$p_{2}^{*}=c_{2}+\frac{2t\left[1-F\left(\frac{p_{2}^{*}-p_{1}^{*}}{2t}+\frac{1}{2}\right)\right]}{f\left(\frac{p_{2}^{*}-p_{1}^{*}}{2t}+\frac{1}{2}\right)}.$$

It follows that the equilibrium price difference is given by:

$$p_{2}^{*}-p_{1}^{*}=c_{2}-c_{1}+\frac{2t\left[1-2F\left(\frac{p_{2}^{*}-p_{1}^{*}}{2t}+\frac{1}{2}\right)\right]}{f\left(\frac{p_{2}^{*}-p_{1}^{*}}{2t}+\frac{1}{2}\right)}.$$

Using *F*(*b*) = *b* ^2^ and consequently also *f*(*b*) = 2*b* we then get:

$$p_{2}^{*}-p_{1}^{*}=c_{2}-c_{1}+\frac{2t\left[1-2{\left(\frac{p_{2}^{*}-p_{1}^{*}}{2t}+\frac{1}{2}\right)}^{2}\right]}{2\left(\frac{p_{2}^{*}-p_{1}^{*}}{2t}+\frac{1}{2}\right)}.$$

To simplify notation, let us use Δ = *p*_2_* - *p*_1_* and *z* = *c*_2_ - *c*_1_. Some algebra then yields:

Δ = [2*t* ^2^/(Δ+*t*)] + *z* - Δ - t. Further algebraic operations give us the equation:

2Δ^2^ + Δ(3*t* - *z*) - *t* ^2^ - *zt* = 0. Using the quadratic formula for the roots of this quadratic equation in Δ then gives the expression in the proposition.

In order to better visualize how the different parameters affect the equilibrium, Table 2 presents the equilibrium price difference for various values of *t* and *z*.

**Table 2:** Equilibrium price difference (Δ = *p*_2_* - *p*_1_*) for *F*(*b*) = *b* ^2^ and various values of *t* and *z* = *c*_2_ - *c*_1_.

*t*	0.25	0.5	0.75	1	2
***z = c***_**2**_ **– *c***_**1**_					
**-2.8**	-0.203	-0.313	-0.354	-0.353	-0.190
**-2.6**	-0.199	-0.300	-0.331	-0.323	-0.144
**-2.4**	-0.195	-0.285	-0.307	-0.291	-0.098
**-2.2**	-0.190	-0.269	-0.279	-0.256	-0.049
**-2**	-0.184	-0.250	-0.250	-0.219	0.000
**-1.8**	-0.176	-0.229	-0.218	-0.180	0.051
**-1.6**	-0.167	-0.204	-0.183	-0.139	0.102
**-1.4**	-0.157	-0.177	-0.145	-0.095	0.156
**-1.2**	-0.143	-0.145	-0.104	-0.049	0.210
**-1**	-0.125	-0.110	-0.060	0.000	0.266
**-0.8**	-0.102	-0.069	-0.012	0.051	0.322
**-0.6**	-0.073	-0.024	0.038	0.105	0.380
**-0.4**	-0.035	0.026	0.093	0.161	0.440
**-0.2**	0.013	0.081	0.150	0.220	0.500
**0**	0.070	0.140	0.211	0.281	0.562
**0.2**	0.137	0.205	0.274	0.344	0.624
**0.4**	0.211	0.273	0.341	0.409	0.688
**0.6**	0.291	0.346	0.410	0.477	0.753
**0.8**	0.375	0.421	0.482	0.547	0.819

Comment to the table: The value in each cell is the equilibrium price difference (Δ = *p*_2_* - *p*_1_*) for the value of *t* shown above and the value of *z* = *c*_2_ - *c*_1_ shown to the left, when the consumer distribution is given by *F*(*b*) = *b* ^2^. The values of *z* were chosen such that the table will be divided roughly equally between positive and negative values of the price difference. Only one value of the two solutions to the equation in Proposition 6 is relevant and is presented in the Table. The second solution has a price difference that is too large to be sustained in equilibrium.

We can see in the table several interesting patterns. First, we see that the equilibrium price difference (Δ = *p*_2_* - *p*_1_*) increases as the cost difference (*z* = *c*_2_ - *c*_1_) increases. That is, an increase in the cost of firm 2 compared to firm 1 also increases the price of firm 2 compared to firm 1. This makes sense because a higher cost increases the optimal price to charge. However, we also see that the price difference changes much more slowly than the cost difference: the cost difference between two adjacent rows is 0.2 whereas the numbers in the cells change by much less than 0.2 from one row to the next. From the top to the bottom row the cost difference changes by 3.6 (-2.8 versus +0.8), whereas the price difference changes by about 0.58 (for *t* = 0.25) to 1.01 (for *t* = 2). The intuition why the price differences are smaller than the cost differences is the following. Consider for example a change in the cost difference that occurs due to one firm's cost increasing while the other firm's cost is unchanged. The firm with the increased cost will lose many customers if it increases its price by the entire increase in its cost, when it faces a competitor whose cost does not change. Therefore the firm's optimal price change after an increase in its cost is to raise its price but by less than the cost change, resulting in the price difference being lower than the cost difference. Similarly, when a firm's cost goes down, the firm is better off using its cost improvement partly to increase its markup per unit and partly to increase its market share than going back to its former markup by lowering the price by the entire cost reduction, and therefore once again not all of the cost change will be reflected in a price change.

Another result we can see is that at some point the sign of the price difference changes. This means that although we have a consumer distribution that gives a strong advantage to firm 2 (because 75% of the consumers are closer to it than to firm 1), which results (in the case of symmetric costs) in a higher price for firm 2, if the cost of firm 2 is sufficiently lower than the cost of firm 1 (*z* being negative with a high absolute value), we can get an equilibrium where the price charged by firm 2 is lower than that of firm 1. In this case, the effect of the lower cost of firm 2, which acts to lower its price, dominates the opposite effect of its higher demand, which acts to increase its price (relative to firm 1's price). The point at which the sign of the price difference changes (i.e., where the cheaper firm becomes more expensive than the other) is different for the different values of *t*. For the *t* values used in Table 2, the switching point is roughly between *z* = –0.3 for *t* = 0.25 and *z* = –2 for *t* = 2. Notice that the switching point is always in a negative value of *z*. The reason is this: the switching point is the point where the firms have equal prices, and this occurs when the tendency of firm 2 to charge higher prices (due to its stronger demand) is offset by its lower cost, i.e., *c*_2_ < *c*_1_, which by definition implies *z* < 0.

When we look at the effect of *t* on the equilibrium price difference we see that the result we had in the main model, namely that the price difference is proportional to *t*, occurs for the case of symmetric costs (the row of *z* = 0) but not elsewhere. In addition, the effect of *t* is not always monotonic: for example, when *z* = –1.4 we have the most negative price difference for *t =* 0.5 (rather than for one of the extreme values of *t* in the table, 0.25 or 2), and similarly for *z* = –2.4 we have the most negative price difference for *t* = 0.75.

## Conclusions

Given the wide use of the linear-city framework, it seems important to analyze its implications with a general (unlimited to a particular functional form) consumer distribution. The article does so and develops a model that can be useful as a tool for further development by other researchers interested in applying the framework for the analysis of various topics. The model also allows us to analyze the case of asymmetric consumer distribution, which is important because in real markets this distribution can often be asymmetric. Moreover, the model yields equilibrium price difference (when the consumer distribution is asymmetric) and thus allows us to use the linear-city framework to analyze equilibrium price differences. The results suggest that the equilibrium price difference is proportional to the transportation cost parameter, and does not depend on the good's cost. The firms' markups are also proportional to the transportation cost. Another result states the two firms’ prices will be equal in equilibrium if and only if half of the consumers are located to the left of the city’s midpoint; notice that the consumer distribution can still be highly asymmetric. Finally, an extension analyzes what happens when the firms have different costs and how the two sources of asymmetry—the consumer distribution and the cost per unit—interact together.

Several directions for future research that can use the model presented here and add to it are to analyze a model in which firms choose locations before the pricing stage; to adopt different demand functions, for example where some consumers may buy more than one unit; and to examine in more detail the model with specific forms of consumer distributions.
